# A web-based multi-genome synteny viewer for customized data

**DOI:** 10.1186/1471-2105-13-190

**Published:** 2012-08-02

**Authors:** Kashi V Revanna, Daniel Munro, Alvin Gao, Chi-Chen Chiu, Anil Pathak, Qunfeng Dong

**Affiliations:** 1 Department of Biological Sciences, University of North Texas, Denton, Texas, 76203, USA; 2 The Texas Academy of Mathematics and Science, University of North Texas, Denton, Texas, 76203, USA; 3 Department of Computer Science and Engineering, University of North Texas, Denton, Texas, 76203, USA

## Abstract

**Background:**

Web-based synteny visualization tools are important for sharing data and revealing patterns of complicated genome conservation and rearrangements. Such tools should allow biologists to upload genomic data for their own analysis. This requirement is critical because individual biologists are generating large amounts of genomic sequences that quickly overwhelm any centralized web resources to collect and display all those data. Recently, we published a web-based synteny viewer, GSV, which was designed to satisfy the above requirement. However, GSV can only compare two genomes at a given time. Extending the functionality of GSV to visualize multiple genomes is important to meet the increasing demand of the research community.

**Results:**

We have developed a multi-Genome Synteny Viewer (mGSV). Similar to GSV, mGSV is a web-based tool that allows users to upload their own genomic data files for visualization. Multiple genomes can be presented in a single integrated view with an enhanced user interface. Users can navigate through all the selected genomes in either pairwise or multiple viewing mode to examine conserved genomic regions as well as the accompanying genome annotations. Besides serving users who manually interact with the web server, mGSV also provides Web Services for machine-to-machine communication to accept data sent by other remote resources. The entire mGSV package can also be downloaded for easy local installation.

**Conclusions:**

mGSV significantly enhances the original functionalities of GSV. A web server hosting mGSV is provided at http://cas-bioinfo.cas.unt.edu/mgsv.

## Background

Identifying conserved genomic features (*e.g.*, genes) as well as their relative ordering in genomes (*i.e.*, synteny) is important in comparative genomics to understand genome evolution and pinpoint functionally important genomic elements [1]. Computational tools have been actively developed for synteny identification, *e.g.*, MCScan [2] and I-ADHoRe [3]. However, since patterns of genome conservation and rearrangements can be very complicated, visualization tools are critical to reveal those patterns. A variety of web-based synteny visualization tools exist for this purpose (*e.g.*, Ensemble SyntenyView [4], NCBI’s Map Viewer [5], mVISTA [6], SynBrowse [7], GBrowse_syn [8], and CoGe [9]; also see an excellent review in [10]). Compared to standalone bioinformatics software, those web-based analysis tools are more convenient for users since no local software installation or maintenance is necessary. In addition, web-based tools allow users to easily share their results with others (*e.g.*, collaborators in different institutions or the research community in general) by simply sending the web URLs of the result pages. Such result-sharing capability is particularly critical for synteny analysis since other people may need to see the results for themselves with the same view of the data. Using standalone software, different users have to install the same software and load the same data set in their local computers, which may be impractical in many situations. For example, the data is too large to be efficiently distributed via Internet, or the software installation requires a special system some biologists do not have (*e.g.*, Linux).

Although the existing web-based synteny visualization servers are of great value to the research community, they cannot always satisfy biologists’ growing needs. Except for the CoGe server (which is further discussed below), none of the other existing web-based tools allow users to upload their own data for synteny visualization (the mVISTA server allows users to upload genomic sequences for similarity comparison, but it is not designed for explicitly displaying synteny and the accompanying genomic annotation). Instead, those tools often only allow users to analyze a small number of pre-selected genome sequences available at those web resources. This limitation is becoming a serious issue since biologists often need to examine synteny for their own sequences of interest that are typically not available at those web resources. This scenario is already a reality because individual biologists can now sequence a large variety of species, strains or specific genomic regions of interest using the latest generation of DNA sequencing technologies that are accessible to them (*e.g.*, local sequencing facility). Centralized web databases normally do not have the resources for displaying such highly individualized datasets to satisfy users’ diverse needs.

Therefore, we have recently published a web-based genome synteny viewer (GSV), which enables biologists to upload their own synteny and annotation datasets for synteny analysis [11]. However, GSV can only display synteny between two genomes, which makes it difficult to keep up with the demand of biologists to compare multiple genomes. In this study, we have extended GSV into a multi-Genome Synteny Viewer (mGSV). Similar to GSV, mGSV is a web-based tool that allows users to upload their own synteny and annotation data files for visualization, but multiple genomes can now be displayed in a single integrated view using either of two viewing modes. Besides serving users who manually interact with the web server, mGSV also provides Web Services for machine-to-machine communication to accept data sent by other remote resources. The entire mGSV package can also be downloaded with easy local installation.

## Implementation

mGSV was implemented with free open-source software under Linux environment. PHP (http://www.php.net) and MySQL (http://www.mysql.com) were used to implement the web interface and backend database, respectively. JavaScript, jQuery (http://jquery.com) and Raphael (http://raphaeljs.com/) were extensively embedded in the PHP code for interactive browsing features.

### Input data

A mandatory genome synteny data file is required to use mGSV along with an optional genome annotation data file: both are tab-delimited text files as described in the original GSV package [11]. The synteny data file allows users to specify the genomic location of each conserved region in each pair of genomic sequences. One noteworthy characteristic of the synteny data file is its open-ended format, in which users can provide additional information such as alignment score or percentage of similarity or identity to characterize each of the conserved regions. Such additional information can be used for selecting regions of interest for visualization in the synteny browser described below. An optional genome annotation file can also be submitted to list the accompanying genomic features (*e.g.*, genes) to be displayed as annotation tracks along with the reference genomes. Users can define how each feature is displayed, *i.e.*, the shape and color of each annotation track, in the annotation file. Such display settings can also be dynamically changed in the synteny browser. In addition, if an HTML-style hyperlink is provided to annotate the feature name, then clicking on that feature in the synteny display will open the URL in another tab. This would be useful to link to external information about a particular genomic feature. The mGSV/GSV data formats were developed because none of the existing formats can achieve the above goals easily. Additional details about the input file format are available at http://cas-bioinfo.cas.unt.edu/mgsv/tutorial.php. The input files can be either submitted in plain text or in compressed format (*i.e.*, .zip or .gz) to facilitate fast file uploads in mGSV. Besides file upload, the input files can also be accessed through a user-specified URL.

### Backend database

After users have submitted their data through mGSV, the data will be stored in a MySQL relational database as described in Revanna *et al.*[11]. For each submitted dataset, a separate set of synteny and annotation tables is created so that different datasets are stored separately without interfering with each other. Such design allows the backend database structure to easily support the “open-ended” format of the synteny file. For example, if a synteny data file is uploaded with additional columns such as “score”, “evalue” or any other columns, the dynamically generated database *synteny* table will contain these additional columns as fields, specifically matching the columns in the submitted data file. Additional details about the mGSV database architecture can be found in the *ARCHITECTURE* file in the mGSV downloadable package.

### Synteny browser

**Figure 1:**
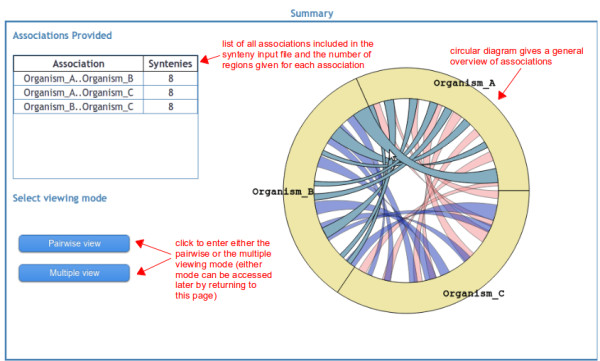
**Summary page.** Upon submission of input files to mGSV, the user is brought to the summary page. A table shows a list of genome associations that were included in the synteny input file, and the number of regions given for each genome pair. A circular diagram gives a general overview of the associations. For the full synteny display, the user can choose to enter either the pairwise or multiple viewing mode.

After the data upload, users are first presented with a summary page (Figure 1), in which all the input genomes are arranged in a circle showing the overall conserved regions among each other (similar to how the Circos software, a standalone visualization tool, can be used for visualizing multiple genome sequence comparison; http://www.circos.ca). An “Associations Provided” chart is also shown in the overview page listing all pairs of genomes specified in the user-uploaded input data and the number of conserved regions for each pair. Below this chart are buttons allowing the user to select either the pairwise or multiple viewing mode.

**Figure 2:**
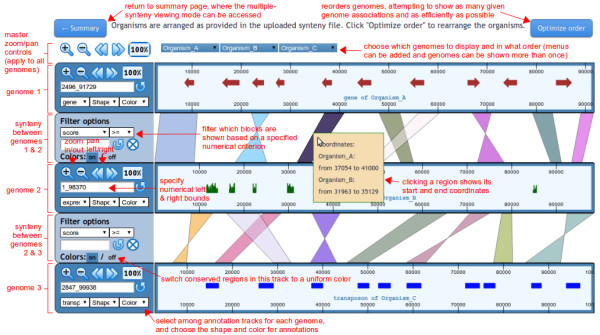
**Pairwise viewing mode.** In this viewing mode, conserved regions are shown between adjacent genomes. Genomes can be rearranged, removed, or shown more than once. Clicking the “Optimize order” button at the top rearranges the genomes to show more associations with fewer total genome tracks. Genome control panels on the left side of the interface allow the genome viewing ranges to be adjusted. Master controls at the top apply to all genomes. If an optional annotation input file was provided, then one annotation track will be shown within each genome track. Using the control panel on the left, users can choose the visible annotation track as well as the shape and color of the annotations. Visible synteny regions can be filtered based on numerical criteria specified for each region in the synteny input file. See main text for more details.

The pairwise viewing mode displays the conserved genomic regions between adjacent genomes (Figure 2). At the top of the synteny browser, multiple pull-down menus are available that allow users to select specific genomes to display in the order of their choice. Additional pull-down menus can be added and removed, so that each genome can be displayed more than once if necessary. For example, to compare a genome *A* to three other genomes *B*, *C*, and *D*, the display can be ordered as ‘*B*-*A*-*C*-*A*-*D’* by using the multiple pull-down menus. Note that there is no designated “reference” genome, which allows for any order of the displayed genomes under the full control of users. Buttons at the top left corner allow users to control all the genomes displayed by zooming in/out, moving left/right or viewing entire genomes on all genomes. mGSV is then divided into two main display windows with control panels (for zoom and filtering functions) on the left and synteny displays on the right. In the synteny display window, each selected genome is represented as a horizontal ruler with tick marks showing its genomic position. The conserved regions between adjacent pairs of selected genomes are displayed as colored translucent blocks. When users click on a conserved region, a pop-up menu appears showing its numerical start and end positions. Users can zoom in/out, move left/right or select specific regions on individual genomes for display by using the embedded control panels on the left of the view. Users can also filter the conserved regions based on their associated characteristics listed in the synteny files such as length of the conserved regions, similarity score, and so on. Selecting and filtering allow users to focus on the regions of interest that meet certain criteria. For example, by applying a stringent similarity cutoff users can choose to only display highly conserved regions. By default, each conserved region is colored differently, but users can change all the displayed regions in a synteny track to be uniformly colored via the *Colors* option. If an annotation file is also provided, a selected annotation track (*e.g.*, gene) will be displayed inside each selected genome. If multiple types of annotations are provided in the annotation file (*e.g.*, both gene and expression profile), only one track per genome can be displayed at a time to avoid overcrowding the display in the current implementation. However, users can easily switch among the tracks or change the colors and shapes of the selected tracks on the fly.

**Figure 3:**
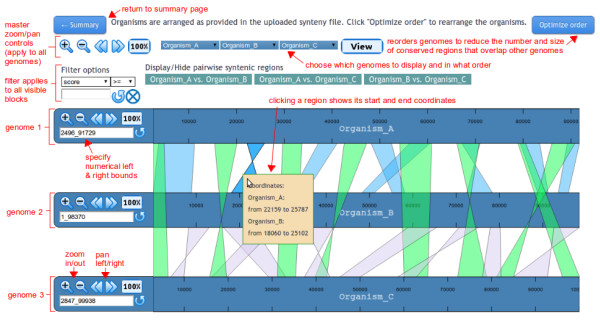
**Multiple viewing mode.** In this viewing mode, conserved regions connecting all visible genomes are shown. The regions associated with one or more particular genome pairs can be hidden using the buttons above the synteny display. Genomes can also be rearranged or removed. Clicking the “Optimize order” button at the top rearranges the genomes to reduce the number and size of conserved regions overlapping other genomes. In this viewing mode, annotations are not shown and each genome is displayed no more than once. As in the pairwise viewing mode, genome control panels allow the viewing ranges to be adjusted. All visible synteny regions can be filtered according to numerical criteria specified in the synteny input file. See main text for more details.

The multiple viewing mode (Figure 3) differs from the pairwise viewing mode in several ways. Most importantly, by default conserved genomic regions are shown among all displayed genomes, rather than just between adjacent tracks. The display of the conserved regions between any pair of genomes can be switched on and off, by clicking on the highlighted synteny pairs displayed above the synteny view. Any genome can be included or removed from the display, provided that each genome is shown only once. Because of the overlapping nature of the conserved region blocks in this view, genome annotations are not shown. The conserved regions in each synteny track have the same color so that overlapping regions can be discerned. All conserved regions can be filtered using a single filter panel above the synteny view.

As mentioned above, mGSV provides two different viewing modes because each method has its own advantages to address specific needs of the user. For example, when many conserved regions are shown, the pairwise viewing mode can appear less crowded. It also allows genome annotations to be shown in each genome block. Users have more control over filtering in this mode, since each synteny track can be filtered independently. On the other hand, the multiple viewing mode can show synteny for more genome pairs in the same viewing window, since all pairwise combinations of the on-screen genomes can be visible. An example of preference to this compactness is seen with just a few genomes allowing the user to visualize all the conserved regions in the same screen. Because the conserved regions for any pair of genomes can be selectively turned off, even complex patterns may be explored in this mode.

### Improving the genome display order

By default, mGSV displays the genomes in the same order as they are specified in the user-supplied synteny files. However, such order may not always be optimal, *i.e.*, the display of the conserved regions may be improved if different adjacent genomes are chosen. Although users can manually adjust the order, we have developed greedy heuristic algorithms for both the pairwise and multiple viewing modes. The algorithmic details are described in the additional file [see Additional file 1]. Although the algorithms do not guarantee to always generate the most optimal orders, they can be used for improving visual clarity by re-arranging the order of the adjacent genomes based on the total size of the conserved genomic regions between each genome pairs. The algorithms involve graph theory and sorting techniques, thus they can be time consuming for datasets with many genomes (*e.g.*, hundreds of genomes, which are unsuitable for being visualized manually anyway). If the optimization can be done rapidly (*i.e.*, before the PHP server times out), the button “Optimize order” will appear in the synteny browser as an option for users to select (Figure 2 and 3).

### Web service for machine-to-machine communication

Besides allowing users to manually specify input files to the mGSV web server, we have also implemented a Web Service to allow machine-to-machine communication so that other programs (*e.g.*, remote bioinformatics databases) may automatically send input data to the mGSV server and obtain the results. The mGSV Web Service is based on standard SOAP specification (http://www.w3.org/TR/soap/). A standalone server application is implemented in Java and runs alongside the mGSV web server as a background process. The Web Service listens to the port 8081 for the requests from remote client programs, which make standard XML-SOAP requests by providing either the synteny and annotation data or URLs pointing to the data files. After receiving the data, the Web Service responds back with a unique ID, which can be used to access the visualization results in the mGSV web server. The binary and source files for both the server and client programs are distributed in the mGSV downloadable version with a detailed documentation on their installation and usage. The documentation also includes the mGSV Web Service protocol specification that is provided as a WSDL file (http://www.w3.org/TR/wsdl).

### Utility programs

The mGSV package also provides scripts for converting outputs of BLAST [12] and BLASTZ [13], as well as GFF3 (http://gmod.org/wiki/GFF3#GFF3) format files into mGSV input files.

### Additional features

If the user submits an email address, an email will be sent immediately to the user with two URLs. One URL links to the current mGSV submission, and the other URL is the access to all the results associated with that email address obtained in the last sixty days.

## Results and discussion

The rapid advances of DNA sequencing technologies are enabling biologists to generate genomic sequences from a variety of species or strains. Analysis of the systemic relationships among those genomic sequences is often important, yet the available synteny visualization tools associated with centralized web databases often do not provide the flexibility for displaying highly individualized data. Therefore, we had developed the GSV server that allows users to upload their own genomic data files for visualization. However, GSV software can only display synteny between two selected genomic sequences at a time. Often, biologists are interested in comparing more than just two sequences. In this study, we have extended GSV into mGSV.

To our best knowledge, the Genome Evolution Analysis tool (http://genomevolution.org/CoGe/GEvo.pl) in the CoGe package is the only other web-based synteny visualization tool that also allows users to upload their own data for analysis in the context of a genome browser. However, there are some fundamental differences between CoGe and mGSV regarding their designs and implementations. The actual DNA sequences, *i.e.*, genomic sequences from several species that users want to compare are required for using the CoGe server, which identify the conserved genomic regions from the input sequences via the embedded sequence comparison software (*e.g.*, BLASTZ). Although embedding sequence comparison software may facilitate users, we have chosen not to do so in mGSV mainly for three reasons: (1) Sequence comparison among large genomes is not often practical at a web server due to heavy computational demands. Such computations are better carried out off-line. (2) Identification of synteny is a complicated research challenge. It is unrealistic for a centralized web server to decide which software or methods users should use for their data set. In addition, desired results may only be obtained by combining different software in a highly customized way. (3) Sequence comparison is not the only means for synteny identification. Other types of data (*e.g.*, genetic mapping) may also provide synteny information. Therefore, we have decided to leave the synteny identification to users. Instead, mGSV focuses on being a visualization tool to display user-specified conserved regions along with submitted annotations. Users can apply any third-party software to generate conserved regions and gather annotation data. This approach leads to greater flexibility and customization of the displayed information.

For biologists who have no programming skills at all, it may be a hurdle to create mGSV-format data files from the outputs of third-party software. However, we have decided not to provide web-based parsers for format conversion for the following reasons: (1) Although converting any third-party software output into mGSV-format is theoretically easy, in practice we may not be able to cater to all the needs of biologists from various backgrounds with their own research interests. For example, when deriving synteny information from BLAST output files, some users may consider E-value alone, some may prefer bit score, and some may use coverage, while others may consider any combination of these or other attributes available in the BLAST output. Such a decision is highly specific for different researchers and it is not practical for us to develop customized parsers for biologists. In addition, there are many software programs that biologists may use for producing synteny regions and annotation, so we prefer not to pick and choose a few for the community. (2) Nowadays, biologists who are involved in large-scale genome analysis usually either have some basic programming skills or collaborate with people who have basic programming skills. Anyone who has basic programming skills, *e.g.*, collaborators in the local bioinformatics center or computer science department, can easily help biologists reformat any raw outputs produced by other programs into mGSV formats (but developing a customized synteny browser requires much more effort, which is what mGSV is aiming to provide). Our sample Perl scripts for converting BLAST output, BLASTZ output, and GFF3 format data files are meant to be examples to show users how to reformat their data files from other formats.

## Conclusions

Bioinformatics tools must be upgraded to accommodate biologists’ increasing needs for analyzing multiple genomes. mGSV is a web-based synteny visualization tool that enhances the original functionalities of GSV by allowing biologists to upload their own datasets and visualize the synteny among multiple genomes simultaneously in a single integrated view. The novel design and the implementation of mGSV provide the research community with an important alternative to currently available tools.

## Availability and requirements

The mGSV web server can be accessed at http://cas-bioinfo.cas.unt.edu/mgsv. The entire package is also free for local installation as open-sourced software from the web site under the terms of the GNU General Public License (http://www.gnu.org/licenses/gpl.html). mGSV is portable across Linux distributions and compatible with PHP 5.2.6 (or higher version) and MySQL 5.0 (or higher version). The mGSV Web Service requires JDK 1.6 (or higher version). mGSV installation has been tested on Debian Lenny and Ubuntu Lynx. mGSV can be viewed with Firefox 3.6.15, Safari 3.0, Internet Explorer 7.0 and Chrome 11.0.

## Competing interests

The authors declare that they have no competing interests.

## Authors' contributions

QD conceived the project. QD, KR, DM and CC contributed most of the design. CC implemented an initial version of the software for the pairwise viewing mode. KR revised and developed the final version for both the pairwise and multiple viewing modes. AG developed the algorithms for optimizing genome order for display. AP developed the Web Service. QD and DM wrote the manuscript; KR, CC, AG and AP also contributed to the writing. All authors read and approved the final manuscript.

## Supplementary Material

Additional file 1 **A Microsoft Word document for describing the detailed algorithms for adjusting the display order of the genomic sequences[**[14]**].** Click here for file
