# Supplementary material for: A web-based multi-genome synteny viewer for customized data
Source: BMC Bioinformatics. 2012 Aug 2;13:190. doi: 10.1186/1471-2105-13-190 (PMC3430549; doi:10.1186/1471-2105-13-190)
Supplement: Additional file 1 — A Microsoft Word document for describing the detailed algorithms for adjusting the display order of the genomic sequences[[14]]. [file 1471-2105-13-190-S1.docx]

**Greedy heuristic algorithms for improving the genome display order**

**Pairwise viewing mode** This algorithm attempts to minimize the total number of genomes displayed. The pairwise associations (*i.e.*, there exists a conserved genomic region between two genomic sequences) are represented in a weighted graph, with the genomes representing nodes and the associations representing edges. The lengths of the edges, *E*, is the total size of the conserved regions among the genomic pair (*i.e.*, the ending nodes of the edge). The data is stored in a weighted incidence matrix for the graph. The implementation of the algorithm is based on Dijkstra’s algorithm for solving the shortest path problem (Dijkstra, 1959). This algorithm is commonly modified to solve the longest path problem on acyclic graphs as follows:

1. Mark all nodes as unvisited, and the weight, *W,* on all nodes as 0. Set the initial node as the current node.
2. Mark the current node as visited. From the current node, compare the weight on a neighboring node, *W*_n_, with the length of the edge from the current node to the neighboring node, *E*, added to the weight on the current node, *W*_c_.
3. If *W*_c_+*E* > *W*_n_, set *W*_n_ as *W*_c_+*E*.
4. Repeat from step 2 until the current node has no unvisited neighboring nodes, then set a neighboring node as the current node and repeat from step 2.
5. The longest path is the path taken from the initial node to the node with maximum weight, *W*.

This previously existing implementation only searches acyclic graphs, and the graph representing pairwise associations is cyclic in many cases. The following step accounts for this when the previously determined longest path is *N*_1_-*N*_2_-…*N*_n_:

1. If the terminal node in the longest path, *N*_n_, is a neighbor of a node *N*_x_, except *N*_n-1_, then the edge *N*_n_-*N*_xo_ with the longest length, is added to the end of the path.

Since *N*_xo_ is located in the path twice, the path is a cycle. This accounts for the cycle in the graph.

For our pair-wise synteny display problem the above algorithm is applied to display all associations (*i.e.*, edges) with the least number of genomes (*i.e.*, nodes). First, the longest path in the graph, which does not traverse the same node more than once, is found. For example, the longest path in {{A-B}, {B-C}, {B-D}} is A-B-C. Then, the edges in the path are displayed and removed from the graph. For example, from A-B-C, A-B and B-C are removed from {{A-B}, {B-C}, {B-D}} to obtain {{B-D}}. Longest paths are repeatedly removed from the graph in this fashion until the graph is empty, meaning all associations have been displayed.

**Multiple viewing mode** Since each genome only appears once in the multiple view, the display of the conserved regions often overlaps with genomes and other conserved region displays. The algorithm attempts to minimize the overlaps of displays of the conserved regions with genomes, which also reduces the overlaps with other conserved regions. The algorithm sorts the genomes given in the input data file. The implementation is based on the insertion sort algorithm to generate a list of genomes. A new entry *N* is inserted into the already sorted list *N*_1_, *N*_2_,… *N*_n_ as follows:

1. Define an index *i,* 0 ≤ *i* ≤ n. The index *i* represents the position immediately following *N_i_* in the list. The index 0 represents the position preceding *N*_1_.
2. Define j(*i*) as the total size of the conserved regions among elements preceding *i* and the nodes following *i*.
3. Define k(*i*) = ∑ (| *i – s* | - 1) * *l*, where conserved regions between *N* and *N_s_* exist, for all 1 ≤ *s ≤* n, and *l* is the size of this conserved region.
4. Insert *N* into the list at the index *i*_o_ where the value j(*i*_o_) + k(*i*_o_) is minimized for all values of *i*.

Entries are inserted into the list from the greatest number of associated conserved genomic regions to the least. This ensures that the list is centered around the most important genomes as it is being built. This approach does not always produce the optimal layout, although the layout produced will almost always be more orderly than a random layout.

**Reference:**

Dijkstra EW: **A Note on Two Problems in Connexion with Graphs**. *Numerische Mathematik* 1959, **1**:269-271.
